# Emerging Strategies for the Prevention of Chemotherapy-Induced Cardiotoxicity in Paediatric Cancer Patients: Advances and Future Perspectives

**DOI:** 10.3390/ph18111604

**Published:** 2025-10-23

**Authors:** Alice Pozza, Angela Di Candia, Luca Zanella, Emil Joly Stefors, Elena Bennati, Camilla Somigli, Elena Poli, Emmanuelle Fournier, Raphael Joye, Rossella Mura, Franca Fagioli, Nicoletta Bertorello

**Affiliations:** 1 Paediatric Cardiology Unit, Department of Women’s and Children’s Health, University of Padua, 35122 Padova, Italy; 2 Department of General Paediatrics, ASL Foggia, 71121 Foggia, Italy; 3 Department of Medical and Surgical Sciences, Alma Mater Studiorum-University of Bologna, 40126 Bologna, Italy; 4 Department of Paediatric Cardiology, Oslo University Hospital, 0424 Oslo, Norway; 5 Cardiology, Meyer Children’s Hospital IRCCS, 50139 Florence, Italy; 6 Paediatric Residency Program, Department of Women’s and Children’s Health, University of Padua, 35122 Padova, Italy; 7 Department of Congenital Heart Diseases, Reference Center for Complex Congenital Cardiac Disease M3C, Marie Lannelongue Hospital, 92350 Plessis Robinson, France; 8 Paediatric Cardiology Unit, Department of Paediatrics, Gynecology and Obstetrics, Geneva University Hospitals, 1205 Geneva, Switzerland; 9 Paediatric Hematology-Oncology, ARNAS G. Brotzu, 09134 Cagliari, Italy; 10 Paediatric Onco-Haematology Department, Regina Margherita Children’s Hospital, University of Turin, 10124 Turin, Italy

**Keywords:** cancer therapy, anthracyclines, cardiotoxicity, paediatric, childhood cancer survivors

## Abstract

Chemotherapy-induced cardiotoxicity (CIC) represents a major long-term complication in paediatric oncology patients, with conventional cardioprotective agents providing only limited efficacy. As survival rates improve, preserving cardiac function has become essential for ensuring quality of life in childhood cancer survivors (CCS). A multi-modal approach combining pharmacological agents, gene- and RNA-based technologies, cell therapies, and immune modulation holds great potential for long-term cardiac preservation. As paediatric-specific research advances, successful integration of these emerging strategies into standard care will require multidisciplinary collaboration, long-term monitoring, and ethical safeguards tailored to children. This narrative review aims to provide a comprehensive overview of both established and novel strategies for preventing or reducing CIC in paediatric cancer patients, critically examining recent progress, assessing their efficacy and safety, and outlining key priorities for future research and clinical application.

## 1. Introduction

Paediatric cardio-oncology remains an evolving field, with growing attention on refining strategies to detect and mitigate chemotherapy-induced cardiotoxicity (CIC).

Children, who account for roughly 5% of all new annual cancer diagnoses, now achieve 5-year survival rates exceeding 85% [1]. Consequently, an expanding population of childhood cancer survivors (CCSs) is at risk of late-onset cardiovascular complications and treatment-related cardiotoxicity, particularly given the use of anthracyclines (ACs) in many standard regimens [2,3]. Despite these improved survival outcomes and the unique physiological characteristics and susceptibilities of the paediatric developing heart, most current evidence in cardio-oncology is derived from the adult population, highlighting the pressing need to advance research specifically dedicated to paediatric cardio-oncology [4,5].

According to the International Guidelines Harmonization Group (IGHG), CIC in cancer survivors is defined as a dose-related cardiovascular effect that persists even after the causative treatment has ended [6,7]. The mechanisms of CIC are deeply explored, and it is the leading cause of non-cancer morbidity and mortality in CCSs, with impacts on the healthcare system [2,8].

Although each anti-cancer regimen is associated with specific forms of CIC, a unifying mechanism involves oxidative stress mediated by reactive oxygen species and free radicals [9]. The underdeveloped antioxidant defence system in paediatric patients compromises their capacity to mitigate oxidative damage, thereby increasing their susceptibility to treatment-related cardiotoxicity.

The spectrum of oxidative stress leads to lipid peroxidation, protein damage, and apoptosis of cardiomyocytes, resulting in impaired myocardial function. Additionally, chemotherapy can disrupt calcium homeostasis and mitochondrial function, further contributing to cell death and fibrosis. Over time, the loss of functional cardiomyocytes and replacement by fibrotic tissue leads to decreased myocardial contractility, ventricular dilation, and, ultimately, heart failure (HF) [10]. The cumulative dose of cardiotoxic drugs, age at treatment, and genetic predisposition influence the severity and onset of cardiotoxicity in paediatric patients.

The aim of this review is to provide a comprehensive overview of current and emerging approaches designed to detect, prevent, or mitigate CIC in paediatric cancer patients, highlighting the evolution of strategies, ongoing trials, and recent advances; evaluating their efficacy and safety; and identifying future directions for research and clinical practice.

## 2. Biomarkers for CIC Detection

The main purpose of cardiac biomarkers in cardio-oncology is to enhance risk stratification at baseline and to ensure timely identification of CIC, both during cancer therapy and in long-term surveillance [11]. While classical blood-derived biomarkers—cardiac troponin (Tn) and natriuretic peptides (NPs)—are well-established in clinical practice for the detection of cardiac injury and HF, emerging novel biomarkers aim to detect specific CIC pathophysiological processes [12].

### 2.1. Classical Biomarkers

Cardiac Tn serves as marker of acute myocardial injury, and it is recommended in adult cardio-oncology guidelines for monitoring during cancer treatment, as well as after it [4,11]. In children treated with doxorubicin for high-risk acute lymphoblastic leukaemia (ALL), Tn levels increase during the initial 90 days of therapy, and this elevation is associated with cardiac abnormalities detected during follow-up [13]. A meta-analysis found that although Tn increased with AC therapy, there was no clear evidence of it being associated with left ventricular (LV) dysfunction [14]. Recent evidence suggests that the diagnostic performance of Tn is enhanced when assessed in combination with NPs, clinical parameters suggestive of HF, and clinical aspects related to cancer treatment (age, sex, cumulative AC dose, radiation therapy). This integrated approach may improve the ability to rule out LV dysfunction and thereby minimise the need for echocardiographic assessment [15]. Meo et al. showed that high-sensitive analysis might improve the use of Tn in risk stratification and monitoring during treatment [16]. The IGHG guidelines state that Tn exhibits limited sensitivity for the detection of asymptomatic cardiomyopathy and should not be employed as a standalone surveillance strategy [17].

Brain natriuretic peptide (BNP) or the prohormone N-terminal proBNP (NT-proBNP) rise in response to ventricular wall stress and are established markers for HF, incorporated in both adult and paediatric HF guidelines [12]. A meta-analysis found an association between elevated NPs and LV dysfunction in paediatric patients receiving high-dose AC therapy. Given their limited sensitivity, the authors concluded that NPs should serve as an additional tool, to be employed in combination with clinical assessment and echocardiography [14]. More recently, a study found that combining NT-proBNP with abnormal global longitudinal strain (GLS) can help to identify CCSs at risk of developing future cardiomyopathy (four-fold-increased hazard). When a cumulative AC dose of ≥250 mg/m^2^ is considered, the risk rises to a 14-fold increase [17]. IGHG guidelines conclude that assessment of BNP/NT-proBNP levels might be useful in association with imaging, particularly because, despite its limited sensitivity, it demonstrates high specificity in detecting clinically relevant cardiotoxicity [18].

The role of classical biomarkers in the paediatric setting is not supported by large trials, and their independent prognostic value remains uncertain; therefore, efforts are directed toward integrating them with novel molecules.

### 2.2. Emerging Biomarkers

Detecting CIC before irreversible damage occurs remains an unmet need. Emerging circulating biomarkers, reflecting the complex interplay of oxidative stress, apoptosis, inflammation, and fibrosis, hold promise for transforming early diagnosis and enabling personalised management. However, despite encouraging preclinical and adult data, robust paediatric-specific evidence is lacking, thus limiting immediate clinical translation to this cohort.

Circulating miRNAs (miR-34a, miR29a, miR-126, miR423, miR-499) have been identified as epigenetic regulators of many specific cellular processes, such as angiogenesis, apoptosis, and cardiac cell contractility [19]. Preclinical models and adult observational studies demonstrated their potential to provide pathomechanistic information, acting as markers of oxidative stress, apoptosis, fibrosis, and metabolic shift. Nevertheless, the clinical applicability of miRNAs as early biomarkers is hindered by variability in expression patterns and limited validation in paediatric cohorts [20,21]. Current evidence remains largely preclinical or derived from adult studies, underscoring the need for dedicated paediatric research to define diagnostic thresholds and prognostic values.

Complementing the regulatory role of circulating miRNAs, protein biomarkers provide measurable endpoints of cardiac injury. In adult breast cancer patients, Myeloperoxidase (MPO), an enzyme released during oxidative stress and extracellular matrix degradation, has been correlated with a decline in left ventricular ejection function (LVEF) [22,23]. As such, it could be a possible biomarker for the early detection of cardiac dysfunction, with improved performance when combined with high-sensitivity cardiac Tn I (hs-cTnI) [24]. However, these findings stem predominantly from adult cohorts and preclinical models, with no direct paediatric data available. While promising as demonstrated in a recent animal trial, MPO’s role as a pharmacologic target remains speculative without prospective paediatric trials [25].

In addition to MPO, cytokines like growth differentiation factor-15 (GDF-15) and soluble suppression of tumorigenicity 2 (ST-2) have emerged as sensitive markers of myocardial strain and inflammation. Their levels correlate with AC-induced cardiac injury in adults and some paediatric observational studies [26,27,28]. The prognostic accuracy and clinical utility require validation through randomised control trials (RCTs), which are currently lacking.

Markers reflecting angiogenesis and fibrosis, such as placental growth factor and galectin-3, have been implicated in CIC [20,29,30,31]. Adult studies and preclinical data suggest these biomarkers may identify patients at risk of cardiac dysfunction, but evidence in paediatric population is limited.

Additionally, advances in proteomics and metabolomics identified 27 proteins predictive of severe AC-induced cardiomyopathy in CCS by using an untargeted mass spectrometry approach [32]. While representing a step towards precision medicine, these findings require prospective multicentric validation in the paediatric cohort.

As we are moving into the era of personalised medicine, the potential role of artificial intelligence to individualise surveillance and integrate multi-omics models with clinical data was highlighted by Khera et al. in the America Heart Association scientific statement [33]. Besides customising the approach, this strategy might enhance the understanding of the pathophysiology of CIC, enabling identification of complex biomarker signatures predictive of subclinical cardiac dysfunction, opening the way for individualised cardio-oncology care (Table 1).

The utility of both classical and emerging circulating cardiac biomarkers in paediatric cardio-oncology appears promising; nevertheless, the evidence is still limited considering the predominance of adult data and preclinical findings.

## 3. Emerging Therapeutic Approaches to Treat CIC

While conventional pharmacological strategies remain a cornerstone of cardio protection, a new wave of targeted molecular therapies is reshaping the landscape offering more precise interventions.

Nowadays, dexrazoxane is approved by both the Food and Drugs Administration (FDA) and European Medicines Agency (EMA) for the primary prevention of AC-induced cardiotoxicity in paediatric patients [34]. Past literature has reported concerns regarding the increased risk of secondary malignancies in children treated with dexrazoxane [8,35,36]. It functions as an iron chelator and topoisomerase IIβ inhibitor, thereby reducing oxidative stress and DNA damage in cardiomyocytes, without impairing anti-cancer activity [37,38,39]. Its efficacy in reducing cardiac toxicity, reflected by an elevation in TnT, has been demonstrated in children with high-risk ALL [40]. After a reanalysis of the available data by the Committee for Medicinal Products of Human Use, in 2017, the EMA authorised the use of the drug only in children aged 0–18 years receiving cumulative doses of ≥300 mg/m^2^ of doxorubicin or equivalent, lifting previous restrictions [36]. To date, IGHG guidelines recommend cardio protection with dexrazoxane when the cumulative doxorubicin dose is at least 250 mg/m^2^ [38]. In adults, dexrazoxane is indicated in patients with advanced and/or metastatic breast cancer who have received a prior cumulative dose of 300 mg/m^2^ of doxorubicin or 540 mg/m^2^ of epirubicin and who are candidates for further AC treatment. This drug is endorsed in the current European Society of Cardiology (ESC) and National Comprehensive Cancer Network guidelines for high-risk paediatric patients [4]. The recommendations drawn up in the Australian and New Zealand Delphi Consensus are more liberal in the use of dexrazoxane in the paediatric population [41]. Dexrazoxane should be used in patients at high risk for cardiotoxicity, defined by those aged < 5 years old at diagnosis, in cases of combined therapy with a cumulative dose of ≥100 mg/m^2^ of AC and thoracic radiotherapy of ≥15 G and in cases of a total cumulative AC dose of ≥250 mg/m^2^ [17,18].

To prevent AC-induced cardiotoxicity, different infusion schedules have been studied. A Cochrane review suggested that infusion durations of six hours or longer significantly reduced the risk of clinical HF compared with shorter durations, without evidence of reduced clinical response [42]. However, most patients considered in the study were adults and limited data were available for children. In the paediatric setting, Berrak et al. found that continuous AC infusion was associated with a lower incidence of clinically evident cardiac dysfunction compared with bolus administration [43]. In contrast, Lipshultz et al. demonstrated no significant clinical benefit of continuous infusion, as both treatments resulted in cardiac damage [44]. This discrepancy derives from methodological differences in the study design and suggests that while continuous infusion might reduce overt CIC, it does not eliminate underlying cardiac injuries. Therefore, beyond infusion protocol changes, additional cardioprotective strategies are mandatory.

Further attempts to reduce AC-induced cardiotoxicity brought on the development of liposomal preparations in which conventional AC was encapsulated in liposomes, which drive the AC inside the tumour rather than to the cardiac cells [42].

De Baat et al. systematically reviewed the available evidence on the use of liposomal AC in childhood cancer patients, highlighting its potential benefits in reducing CIC risk [45]. Randomised clinical trials have further explored this approach in paediatric acute myeloid leukaemia (AML). In the AML-BFM 2004 study, liposomal daunorubicin was compared with idarubicin as an induction therapy, showing comparable efficacy and acceptable toxicity profiles [46]. Moreover, the international BFM Study Group trial in relapsed paediatric AML demonstrated improved early treatment response and survival with liposomal daunorubicin added to fludarabine, cytarabine, and granulocyte colony-stimulating factor (FLAG/DNX), suggesting that this formulation may offer both therapeutic efficacy with a favourable cardiotoxicity profile [47]. Nevertheless, although liposomal ACs offer a promising approach to reduce cardiotoxicity, clinicians should remain attentive to other potential adverse effects and consider an individual patient’s risk profile when tailoring treatment.

Clinical HF, either during or after cancer therapy, is managed according to the American College of Cardiology/American Heart Association guidelines [48]. Classical HF therapies aim to block either the renin–angiotensin cascade (angiotensin-converting enzyme inhibitors, angiotensin receptor antagonists) or the sympathetic nervous system (beta-adrenoreceptor antagonist) to reduce afterload and avoid adverse remodelling [49]. When used in primary prevention of CIC, HF strategies reached variable results in the paediatric trials [35,37].

Sodium–glucose co-transporter 2 (SGLT2) inhibitors were initially developed as oral antidiabetic agents; however, due to expression of SGLT2 in cardiac tissue, they have demonstrated cardiovascular benefits in the setting of HF with different LVEF [50]. Their cardioprotective effect is mediated through anti-inflammatory, anti-apoptotic, and antioxidative mechanisms with the restoration of intracellular electrolyte homeostasis [51]. The potential cardioprotective role of SGLT2 inhibitors in cancer patients remains unclear, and its specific use in the paediatric setting requires further investigation, addressing the involved molecular pathways [52].

As cardiotoxicity involves a complex interplay of oxidative stress, inflammation, and cell death pathways, multifaceted therapeutic strategies are needed to effectively protect the heart.

A pivotal mechanism in this pathogenesis is oxidative stress driven by NADPH oxidase 2 (NOX2), which not only generates damaging reactive oxygen species (ROS) but also primes sterile inflammation via the activation of the NLRP3 inflammasome. The downstream inflammatory cascade amplifies myocardial injury, highlighting the potential of novel NLRP3 inhibitors such as INF200 to attenuate this detrimental response.

Beyond inflammation, oxidative stress also triggers ferroptosis, a distinct iron-dependent cell death mechanism characterised by lipid peroxidation, particularly relevant in AC cardiotoxicity where iron overload exacerbates damage. Targeting ferroptosis with inhibitors like liproxstatin-1 opens new avenues to preserve cardiomyocyte viability and mitochondrial function [53].

Given the intricate molecular networks involved in cardiotoxicity, RNA-based therapies offer a precise approach to modulate gene expression, silencing pro-death or pro-inflammatory genes while enhancing survival pathways. For example, small interfering RNA (siRNA) can target p53 or inflammatory cytokines. Additionally, messenger RNA (mRNA) can encode protective factors such as VEGF or sirtuin 1 (SIRT1). These technologies exemplify how to complement oxidative and inflammatory inhibition strategies [29,54].

Building on gene modulation, stem cell therapies—notably mesenchymal stem cells (MSCs) and induced pluripotent stem cells (iPSCs)—contribute through paracrine effects and tissue regeneration, while gene editing tools like CRISPR/Cas9 enable direct correction or silencing of cardiotoxic mediators (topoisomerase IIβ and genes like SOD2 and PGC-1α), thereby addressing root causes at the DNA level [55,56].

Complementary to molecular interventions, immunotherapy targeting cytokines like IL-1 and TNF-α, or modulating immune cell phenotypes, serve to fine-tune the inflammatory environment, reducing injury and fostering repair [57]. Importantly, these therapies can be tailored to specifically target the inflammatory response within the heart, thereby minimising the risk of systemic immunosuppression and its associated side effects. Emerging cell-based immunotherapies, such as engineered Chimeric Antigen Receptor T cells (CAR-T cells), hold promise for targeted delivery of cardioprotective agents [58]. Enhancing regulatory T cells and shifting macrophage phenotypes from pro-inflammatory to reparative may further reduce inflammation-induced injury while preserving systemic immune function [21].

To enhance delivery and efficacy, exosome-based therapies derived from MSCs provide a novel means to transfer protective RNA and proteins, while mitochondria-targeted antioxidants like MitoQ and SS-31 specifically counteract AC-induced mitochondrial dysfunction [59].

When innovative cancer therapy and paediatrics intersect, ethical challenges and considerations arise. Given that the paediatric heart is still undergoing development within the context of overall somatic growth, children require additional considerations in both the prevention and management of CIC.

As an example, the use of CRISPR in the paediatric field requires stringent oversight because of potential off-target effects in developing cells at risk for genetic editing. Besides being revolutionary in cardio-oncology, CAR-T is associated with cytokine release syndrome and the risk of neurotoxicity with poor outcomes and high morbidity.

Stem cell therapies, despite regenerative promise, face barriers in standardisation, potential tumorigenicity, and immunogenicity. These revolutionary therapies can also be combined, but they require a deep risk–benefit evaluation and transversal adherence to the non-maleficence principles of medical practice [60].

Beyond direct cardiac interventions, systemic factors such as gut microbiota also influence cardiac health, presenting novel opportunities for probiotic or prebiotic therapies, although paediatric evidence is still limited. Meanwhile, targeting cellular stress responses through proteasome and autophagy modulation offers additional layers of cardio protection by preserving cellular homeostasis during chemotherapy [61,62].

Overall, these targeted approaches represent a mechanistically precise arsenal against cardiotoxicity, aiming to intervene at multiple critical nodes to maximise cardio protection while minimising off-target effects. Table 2 outlines a range of therapies used or under investigation to prevent/reduce CIC, including their mechanism of action and representative examples. The incorporation of paediatric-specific cardiac models has the potential to improve a safe and effective translation of innovations to young cancer patients. Importantly, any strategy aimed at protecting cardiac health must be balanced while keeping oncologic effectiveness, and its ultimate adoption should be guided by high-quality clinical studies demonstrating clear benefits in overall survival or quality of life to avoid unintended burdens from premature implementation.

## 4. Ongoing Clinical Trials on Novel Strategies for Preventing or Reducing CIC

A growing number of trials are exploring innovative strategies for early detection and prevention of CIC, increasingly combining advanced imaging with multi-omics approaches to enable precision medicine (Table 3).

The NCT01671696 study targets asymptomatic cardiotoxicity in CCSs using cardiac magnetic resonance (CMR) and serological biomarkers, investigating whether parameters such as circumferential strain and T1 mapping outperform standard echocardiography [63]. In parallel, DNA/microRNA profiling assesses dose-dependent molecular changes. The SpeckleAnthra2 study (NCT05781672), which builds on prior paediatric cohort data demonstrating persistent GLS impairment years after therapy, extends long-term follow-up in paediatric patients treated with AC [64,65]. The study employs speckle-tracking echocardiography to refine long-term risk stratification based on treatment intensity and patient characteristics. CATCH-HF (NCT04262830) further broadens this perspective by applying comprehensive CMR protocols in adolescents and young adults previously exposed to cardiotoxic regimens [66]. The goal is to correlate early imaging abnormalities with structural and functional cardiac changes, improving the detection of preclinical dysfunction.

In adult survivors of paediatric cancers, NCT04852965 focuses on late-onset cardiomyopathy, integrating echocardiography, CMR, strain imaging, and both conventional and emerging biomarkers—including MPO, IL-6, sST2, and microRNAs—to define predictive molecular signatures of cardiotoxicity [67].

Collectively, these studies highlight a paradigm shift toward tailored surveillance strategies. While paediatric-specific evidence is still evolving, this multidimensional approach holds promise for identifying high-risk individuals and guiding timely, personalised interventions.

There is currently limited evidence from randomised studies evaluating how unsupervised physical activity interventions affect cardiovascular health in children undergoing cancer treatment and survivors. Despite increasing recognition of its importance, exercise guidelines tailored specifically for paediatric oncology patients are lacking [68,69,70]. Moreover, the relationship between physical activity levels and cardioprotective effects, documented in the improvement in cardiac biomarkers or MRI findings, remains poorly understood in this group.

Some research, including a Cochrane review and a meta-analysis by Bourdon et al., has shown that exercise may improve fitness and quality of life in CCSs, though findings are limited by methodological inconsistencies [71,72].

Several ongoing studies aim to address these gaps. The HIMALAYAS randomised controlled trial (NCT05023785) evaluates a structured cardio-oncology rehabilitation programme for adolescent and young adult cancer survivors with subclinical cardiac dysfunction, focusing on improving cardiorespiratory fitness (VO_2_ peak, primary outcome) and cardiac health [73].

While CCSs may not experience the same level of cardiovascular benefits from physical activity as their healthy peers—likely due to chemotherapy and radiation-related damage to the heart and blood vessels—the NCT04036032 trial is investigating whether aerobic exercise can still promote meaningful improvements. Specifically, the study explores whether exercise can reverse AC-induced cardiotoxicity through cardiac remodelling assessed via CMR and enhance cardiopulmonary function and changes in molecular markers such as microRNA expression [74].

Finally, the BEACON study is investigating barriers and facilitators influencing adherence to structured physical activity, developing a personalised, evidence-based intervention to promote long-term engagement among CCSs [75].

Altogether, these studies highlight the need for individualised, evidence-based exercise programmes aimed at preventing or reversing treatment-related cardiac damage and improving long-term outcomes in this vulnerable population.

A promising unusual non-pharmacological strategy for preventing AC-induced cardiotoxicity is remote ischemic conditioning (RIC). This technique involves inducing brief periods of restricted blood flow followed by reperfusion in a limb, which has been shown to protect organs, including the heart. Preclinical studies, particularly a pig model with AC-induced cardiomyopathy, have demonstrated RIC’s potential to preserve heart function and protect mitochondria from damage, including the preservation of mitochondrial dynamics and dysregulated autophagy, occurring early during AC-induced cardiomyopathy [76]. Importantly, its protective mechanism differs from those of other treatments, suggesting that it may offer additional, non-overlapping benefits.

Though RIC has shown promise in adult myocardial infarction and stroke, a small paediatric sham-controlled RCT (68 patients, mean age 11 years, with various cancers) showed no significant benefit. The results might have been affected by limitations in the protocol, including low AC exposure, minimal baseline risk of cardiomyopathy in this population, and the use of Tn and echocardiography as markers of dysfunction [77,78]. However, the ongoing RESILIENCE (NCT05223413) trial is targeting high-risk adult lymphoma patients receiving ≥ 5 AC cycles [79]. This trial incorporates advanced cardiac MRI techniques, including T2 mapping and a novel ultrafast cine sequence (which lasts less than 1 min), to detect early cardiac changes and assess RIC’s efficacy more precisely than traditional biomarkers like Tn. RESILIENCE is based on the hypothesis that RIC will lower AC incidence, that CMR T2 mapping will outperform other early markers of AC, and that ultrafast CMR will match conventional methods in clinical utility [80]. The trial focuses on a high-risk group, and its use of sensitive imaging tools addresses key limitations of past studies and may reveal a clear protective benefit of RIC.

Overall, the results of these ongoing trials may be particularly valuable, as they may provide further clinical evidence that is still lacking to support cardioprotective strategies in children and adolescents. Their findings may not only validate the use of advanced imaging and biomarker-based approaches for early CIC detection but also clarify how exercise interventions and innovative techniques such as RIC can be integrated into standard care. Ultimately, these studies have the potential to shape future guidelines, moving the field of paediatric cardio-oncology toward precision medicine, and improve long-term cardiovascular outcomes in CCSs.

**Table 3:** Overview of novel approaches for early detection, prevention, and mitigation of CIC.

Registration Name	Study Design	Inclusion Criteria	Age (Years)	Intervention(s)/Diagnostic Test(s)	Number of Enrolled Patients (Planned or Actual)	Primary Outcome(s)	Additional Outcome(s)	Involved Centres
NCT01671696 [63]	Observational Case–control Cross-sectional	CCSs having received ≥240 mg/m^2^ AC. Complete remission and off chemotherapy for ≥2 years	9–35	CMR Other: Echo, serological biomarkers of inflammation, myocyte injury, extracellular matrix remodelling, apoptosis, and BNP. Phenotype analysis of DNA/microRNA.	80	Changes in T1 Imapping-derived relaxation time and circumferential strain analysis	Changes in serological markers of extracellular matrix remodelling and tissue apoptosis; phenotype analysis of DNA/microRNA.	Connecticut Children’s Medical Centre, Hartford, Connecticut (USA)
NCT05781672 (SpeckleAnthra2) [64]	Interventional	CCSs included in the “SpeckleAnthra” Study. In remission of malignant disease. Discontinued chemotherapy for ≥6 years.	11–27	STE analysis.	134	5-year evolution of LVGLS	LVEF. LV myocardial dysfunction; death secondary to toxic cardiomyopathy; Troponin T, NT-proBNP.	University Hospital, Montpellier (France)
NCT04262830 (CATCH-HF) [66]	Observational Cohort Prospective (3–5 years)	CCS previously treated with AC for cancer. Cancer diagnosis ≥ 2 years prior.	13–39	CMR Other: Accelerometer physical activity monitoring.	150	LVEF	**-**	Rady Children’s Hospital, San Diego, California (USA)
NCT04852965 [67]	Observational Cohort Cross-sectional	CCS having received at least 100 mg/m^2^ of AC.	≥18	24 h Holter ECG; echo; CMR. Others: Serological biomarkers (troponin, NT-proBNP) and novel biomarkers (IL6, MPO, and sST2).	103	Cardiotoxicity (as defined by the BSE and BCOS guidelines)	Levels of hs-TnT and NT-proBNP.	Queen’s University, Belfast (United Kingdom)
NCT05023785 (HIMALAYAS) [73]	Interventional Randomised Open label	≤39 years of age at the time of cancer diagnosis. Received cancer treatment(s) with known cardiovascular risks. Be cancer-free at the time of enrolment. Stage B Heart Failure.	18–45	Supervised CORE (cardio-oncology rehabilitation) model, exercise therapy.	336	Cardiorespiratory fitness (cardiopulmonary exercise test, VO_2_peak) at 6-month follow-up	Cardiorespiratory fitness (CPET, VO_2_peak, ventilatory and anaerobic thresholds, HR recovery). LV systolic and diastolic function (LVEF, GLS). LV hypertrophy. Metabolic profile (lipid metabolism, insulin sensitivity, HOMA-IR). Health-related quality of life.	University Health Network, Toronto, Ontario, Canada
NCT04036032 [74]	Observational Prospective	Long-term CCSs ≥ 9 years of age. Exposed to AC chemotherapy.	9–99	Encourage work out 4 to 5 times a week for 3 months. Exercise support.	65	LV and RV volume and mass assessed by CMR	Cardiopulmonary parameters. Quality of life. MicroRNA expression.	Connecticut Children’s Medical Center, Hartford, Connecticut (USA). Nationwide Children’s Hospital, Columbus, Ohio (USA)
NCT05223413 (RESILIENCE) [79]	Interventional Phase II Randomised Double blind Sham-controlled Prospective Multinational	≥18 years old at first lymphoma diagnosis. Scheduled to undergo ≥ 5 chemotherapy cycles including AC. Pre-chemo LVEF > 40% on screening echo. ≥1 risk factor for developing cardiotoxicity	18–99	Device: RIPC Device: Simulated RIPC (Sham)	608	LVEF assessed by CMR	Incidence of AC cardiotoxicity events. Rate of tumour regression. Change in quality of life. Rate of heart failure hospitalisation. Other: Ability of T2 mapping to predict AC cardiotoxicity versus classical markers (LV strain, cardiac injury biomarkers); to validate a novel ultrafast CMR sequence.	Fundación Centro Nacional de Investigaciones Cardiovasculares Carlos III, Madrid, Spain and other European centres

AC = anthracycline; BCOS = British Cardio-Oncology Society; BNP = ventricular derived brain-type natriuretic peptide; BSE = British Society of Echocardiography; CCSs = childhood cancer survivors; CMR = cardiac magnetic resonance; CPET = cardiopulmonary exercise test; ECG = electrocardiogram; HOMA-IR = homeostasis model assessment insulin resistance, HR = heart rate; hs-TnT: high-sensitivity troponin T; IL6 = interleukin-6; LV = left ventricle; LVEF = left ventricle ejection fraction; LVGLS = left ventricle global longitudinal 2D strain; MPO = myeloperoxidase; NT-proBNP = N terminal-pro-brain natriuretic peptide; RIPC = remote ischemic pre-conditioning; RV = right ventricle; sST2 = soluble ST2; STE = speckle-tracking echocardiography.

## 5. Impact of Exercise

Exercise is increasingly recognised as a non-pharmacological strategy to mitigate AC-induced cardiotoxicity, with stronger evidence in the adult than paediatric population.

Experimental and clinical studies have shown that exercise can mitigate oxidative stress, preserve mitochondrial integrity, upregulate antioxidant enzymes, and enhance endothelial function through nitric oxide bioavailability [81,82].

Randomised trials and meta-analyses in adults demonstrate that structured exercise improves peak oxygen uptake (VO_2_peak), helps preserve LVEF, and reduces overall cardiovascular morbidity [83]. These findings support the recommendations of the American College of Sports Medicine and ESC 2022 guidelines, which emphasise the role of supervised aerobic or combined aerobic–resistance programmes, as part of the Cardio-Oncology Rehabilitation (CORE) model in adults [84].

Such evidence in the paediatric setting is still emerging and is limited to small, mostly single-centre studies and systematic reviews [85,86]. Additional barriers include treatment-related immunosuppression, frequent hospitalizations, and psychosocial factors that further complicate adherence to exercise intervention in this population [70]. Family involvement and age-appropriate approaches are essential for feasibility.

In adults, recommendations converge on ≥150 min of moderate aerobic activity per week combined with resistance training 2–3 times weekly [10,12]. High-intensity interval training is under investigation, with promising results in selected cohorts. In children, adapted prescriptions emphasise shorter sessions (15–20 min initially, progressing to 45–60 min) of moderate intensity (40–70% VO_2_peak or 60–80% max heart rate), and the incorporation of play-based or tele-supervised modalities to enhance adherence [7,11].

Bridging the adult–paediatric gap requires multicentre randomised trials with standardised endpoints, including strain imaging and biomarker-based measures of subclinical cardiotoxicity. Integration of exercise physiologists into oncology teams and systematic inclusion of physical activity in survivorship registries are essential. Consensus recommendations from the Association for European Paediatric and Congenital Cardiology (AEPC), Pan-European Network for Care of Survivors after Childhood and Adolescent Cancer (PanCare), and Children’s Oncology Group (COG) support the inclusion of physical activity in follow-up care. However, these guidelines also highlight a significant gap: the lack of standardised, evidence-based exercise protocols tailored to paediatric cancer survivors [70,87]. Given the robust evidence supporting the adult CORE model, its adaptation and rigorous evaluation in the paediatric setting could provide development of effective standardised rehabilitation programmes. Such translation requires addressing unique challenges in the paediatric setting, including developmental considerations, treatment-related vulnerability and social factors.

## 6. Role of Genetic Testing

Although cumulative dosage and the class of chemotherapeutic drugs are recognised predictors of CIC, individual responses can differ significantly, influenced by both genetic predisposition and environmental factors. In this light, the fields of precision medicine and pharmacogenomics present valuable opportunities to tailor treatment plans, enabling more targeted monitoring strategies, particularly in paediatric patients [88].

Visscher et al. analysed 2977 single-nucleotide polymorphisms (SNPs) across 220 genes related to AC metabolism and transport, identifying genetic variants such as the synonymous rs7853758 in SLC28A3 that influences cardiotoxicity risk, alongside other genes like SLC28A1 and several ATP-binding cassette (ABC) transporters with variable effects [89]. While paediatric studies have highlighted genetic susceptibility to AC-induced cardiotoxicity, in the Atkins C.D. study, the need to extend this research into the adult population has been emphasised [90].

On this basis, Orgil et al. developed a systems genetics framework in mouse models that integrates genome-wide quantitative trait locus (QTL) mapping with transcriptomic data to link genomic regions to cardiotoxicity-related traits, assessed in a sex-specific manner [91]. This approach addresses the complexity of AC toxicity, particularly relevant to paediatric patients where age and sex significantly impact vulnerability [92]. The prioritisation of candidate genes within significant QTL regions was especially rigorous. Using a multi-criteria scoring system, the authors evaluated genes based on the presence of protein-altering variants, evidence of cis-regulation in heart tissue, differential expression in failing human hearts, and relevance to cardiac phenotypes in model organisms. HS3ST4 gene was identified near a novel SNP associated with an increased risk of AC-induced cardiomyopathy and was upregulated in an iPSC-derived cardiomyocyte model from patients who experienced AC-related cardiac injury. In the paediatric population, the developing myocardium is more susceptible to iron-mediated lipid peroxidation and mitochondrial injury [93,94]. Therefore, identification of ferroptosis-related gene networks suggests potential targets for cardioprotective therapies and aligns with emerging evidence that the modulation of these pathways could mitigate long-term cardiac damage.

Interestingly, the Children’s Oncology Group and the Childhood Cancer Survivor Study investigated the role of DNA damage repair (DDR) genes in AC-induced cardiomyopathy [95]. The study explored the role of DDR genes using a case–control design across two large cohorts of CCSs and identified significant associations between AIC and genetic variants in DDR genes, particularly FANCC and XRCC5. A gene–drug interaction was observed with MGMT, where its variants modulated the effect of cumulative AC doses on cardiotoxicity. Functional validation using CRISPR knockouts in human iPSC-derived cardiomyocytes demonstrated that the loss of FANCC or MGMT increased resistance to doxorubicin. The study emphasises the relevance of DDR genes in modulating cardiac vulnerability to AC, as DDR variants may impair or enhance cardiomyocyte DNA repair after drug exposure

Beyond pharmacogenomic research, Garcia-Pavia et al. highlighted the role of TTN mutations in the onset of cardiomyopathy post chemo treatment, and these results were also validated in the study performed by Bennati et al. [96,97]. From a clinical standpoint, genetic testing offers several avenues to enhance current monitoring strategies. First, genetic risk markers could eventually be incorporated into pre-treatment screening panels to stratify patients based on their susceptibility. This would allow clinicians to tailor the use of AC or other cardiotoxic agents, intensify cardiac surveillance, or consider protective agents such as dexrazoxane in a more targeted way. The integration of genetic information may further refine its use, identifying patients who might derive the greatest long-term benefit or who may require alternative strategies.

Despite the promising insights provided by pharmacogenomics and genetic profiling in understanding CIC, several limitations constrain their clinical utility.

Current ESC cardio-oncology guidelines do not recommend routinary use of genetic testing prior to the start of cancer treatment, except for patients with a family history of cardiomyopathy, pre-existing cardiac abnormalities, or those carrying genetic variants associated with cardiovascular disease during cancer treatment [40,85]. This recommendation reflects, at least in part, the high costs, limited clinical applicability of current results, and the complexity of managing data obtained from population-scale genetic analysis. Besides the identification of candidate variants and genes associated with cardiotoxicity risk, the genetic architecture underlying CIC remains incompletely understood. The heterogeneity of the paediatric population itself poses a great challenge for replication of results across different cohorts, limiting the generalizability of findings [98,99].

Additionally, CIC is a multifactorial phenotype influenced by complex gene–gene and gene–environment interactions, which are not fully captured by existing genetic analyses. This complexity hinders the development of comprehensive risk prediction models, predictive algorithms and scoring systems [100].

Consequently, while genetic testing holds great potential to refine CIC risk assessment, further large-scale, multi-ethnic studies are necessary to clarify genetic contributions, validate paediatric risk models, and establish standardised protocols before routine clinical adoption.

## 7. Conclusions

The growing cohort of CCSs highlights the urgent need to address the long-term cardiovascular consequences of cancer therapies. Despite significant advances in paediatric haematology and oncology, paediatric cardio-oncology remains an evolving field, where evidence is often derived from adult studies. While classical biomarkers offer some prognostic value, emerging circulating biomarkers (e.g., miRNAs, MPO, GDF-15, ST2, and galectin-3) show promise in capturing subclinical myocardial injury and specific pathophysiological processes. Novel therapeutic approaches—including RNA-based and immunomodulatory therapies, ferroptosis inhibitors, and exosome-based interventions—underscore a shift toward mechanism-specific and precision-driven cardio protection. Advances in genetic testing and pharmacogenomics further support the development of individualised surveillance and treatment strategies based on patient-specific risk profiles.

Ongoing clinical trials incorporating multi-omics, advanced imaging, and structured exercise programmes point out a paradigm shift from reactive to proactive care [101]. Despite these advances, significant challenges persist in translating novel biomarkers and therapies into age-specific clinical practice.

To overcome these barriers, future efforts must prioritise multicentre trials, age-appropriate models, and the integration of artificial intelligence and Bayesian methods to synthesise complex datasets for real-time decision-making [102,103,104].

Parallel progress in precision medicine, through the incorporation of genomics, pharmacogenetics, and digital health, aims to refine individualised surveillance strategies. Machine learning-driven platforms that integrate imaging, novel biomarkers, and clinical data may enhance risk prediction and reduce the burden of CIC for CCSs. Achieving this successful vision, however, remains contingent on robust multicentric studies involving big survivorship cohorts, and relies on multidisciplinary network across healthcare providers. Prioritising these areas will accelerate the translation of novel biomarkers and mechanism-specific therapies into effective, precision-driven cardiovascular care for CCSs.

## Figures and Tables

**Table 1:** Conceptual framework linking circulating biomarkers, pathophysiological mechanisms, and therapeutic targets in CIC.

Emerging Biomarker	Biomarker Mechanism of Action	Therapeutic Target
Circulating miRNAs	Regulation of apoptosis, angiogenesis, cardiac contractility	Epigenetic modulation therapies targeting signalling pathways influencing cell survival, angiogenesis, and cardiac remodelling
MPO	Oxidative stress, extracardiac matrix degradation	Anti-MPO to reduce oxidative damage and fibrosis
hs-TnI	Cardiomyocyte injury	Cardioprotective agents
GDF-15	Myocardial strain, inflammation	Anti-inflammatory therapies; modulation of stress–response pathways to limit myocardial remodelling
ST-2	Myocardial strain, fibrosis	Anti-fibrotic/anti remodelling agents
PlGF	Angiogenesis	Angiogenesis modulators
Gal3	Cardiac fibrosis and remodelling	Inhibitors of Gal-3, antifibrotic strategies

(Gal3: galectin-3; GDF-15: growth differentiation factor-15; hs-TnI: high-sensitivity troponin I; miRNA: microRNA; MPO: myeloperoxidase; PlGF: placental growth factor; ST-2: soluble suppression of tumorigenicity 2).

**Table 2:** Therapeutic strategies to prevent and manage cardiotoxicity.

Category	Examples	Mechanism/Rationale
Approved primary cardioprotective drugs	Dexrazoxane	Reduces free radicals and DNA damage in cardiomyocytes.
Conventional Drugs	Beta-blockers, ACE inhibitors, ARBs	Support heart function and reduce strain on the heart.
Repurposed Drugs	SGLT2 inhibitors	Lower inflammation and improve heart energy metabolism.
Molecular Targets	NOX2 inhibitors, Ferroptosis blockers	Block oxidative and inflammatory damage to heart tissue.
RNA-Based Therapies	siRNA, mRNA (SIRT1, VEGF)	Adjust gene activity to support heart cell survival.
Stem Cell Therapies	MSCs, iPSC-derived patches	Promote repair and reduce heart scarring.
Gene Editing	CRISPR/Cas9 (Top2β, SOD2)	Switch off harmful pathways linked to heart damage.
Exosome/Mito Therapies	MSC exosomes, MitoQ, SS-31	Deliver protective signals and boost mitochondria.
Gut Microbiota Modulation	Probiotics, Prebiotics	Reduce systemic inflammation
Protein Homeostasis	Proteasome modulators	Maintain healthy proteins and prevent heart cell stress.
microRNA Therapies	miR-146a, miR-21	Fine-tune repair and reduce fibrosis and cardiomyocytes’ death.

ACE: angiotensin-converting enzyme; ARBs: angiotensin receptor antagonist; iPSC: induced pluripotent stem cell; MitoQ: mitoquinone; mRNA: messenger RNA; MSC: mesenchymal stem cell, NOX2: NADPH oxidase 2; SGLT2: sodium–glucose co-transporter 2; siRNA: small interfering RNA; SIRT1: sirtuin 1; SOD2: superoxide dismutase 2; SS-31: Szeto–Schiller peptide; Top2β: topoisomerase II Beta; VEGF: vascular endothelial growth factor.

## Data Availability

No new data were created or analyzed in this study. Data sharing is not applicable to this article.
